# Transvaginal Small Bowel Evisceration following Abdominoperineal Resection

**DOI:** 10.1155/2018/6012809

**Published:** 2018-02-15

**Authors:** Enver Kunduz, Huseyin Bektasoglu, Samet Yigman, Huseyin Akbulut

**Affiliations:** Department of General Surgery, Faculty of Medicine, Bezmialem Vakif University, Istanbul, Turkey

## Abstract

Abdominoperineal resection (APR) is one of the surgical techniques performed for the distal rectal cancer. The perineal herniation is one of the complications of APR surgery. In this report, we aim to demonstrate a rare case of small bowel evisceration and strangulation secondary to the transvaginal herniation evolved in the late stage after perineal hernia repair following laparoscopic APR.

## 1. Introduction

Abdominoperineal resection (APR) is one of the techniques performed in the surgical treatment of the distal rectal cancer [1]. The perineal herniation is one of the complications seen after APR operations [2]. The perineal herniation after APR was described for the first time at the end of the 1930s, and it is not presented in the literature as a series but rather as a presentation of cases [3]. Various techniques such as synthetic patch, reconstruction with the muscle flap, and biomedical patch repair are recommended in the literature to prevent more frequent herniation due to a large perineal defect in the extralevator APR operations [4]. The perineal herniation after classical APR is restored by posterior or abdominal approaches [5]. The vaginal reconstruction is needed in cases of posterior vaginal wall resection due to the posterior wall invasion of the tumor, but the reconstructive techniques are not recommended for vagina in classical APR [6]. Although there have been several reports about the transvaginal herniation following hysterectomy or trauma, we could not find any report of the transvaginal herniation and evisceration following the abdominoperineal resection and the perineal hernia repair in English literature. This article aims to present a case of transvaginal evisceration and small bowel strangulation in a patient who underwent perineal hernia repair after laparoscopic APR.

## 2. Case Report

**Figure 1:**
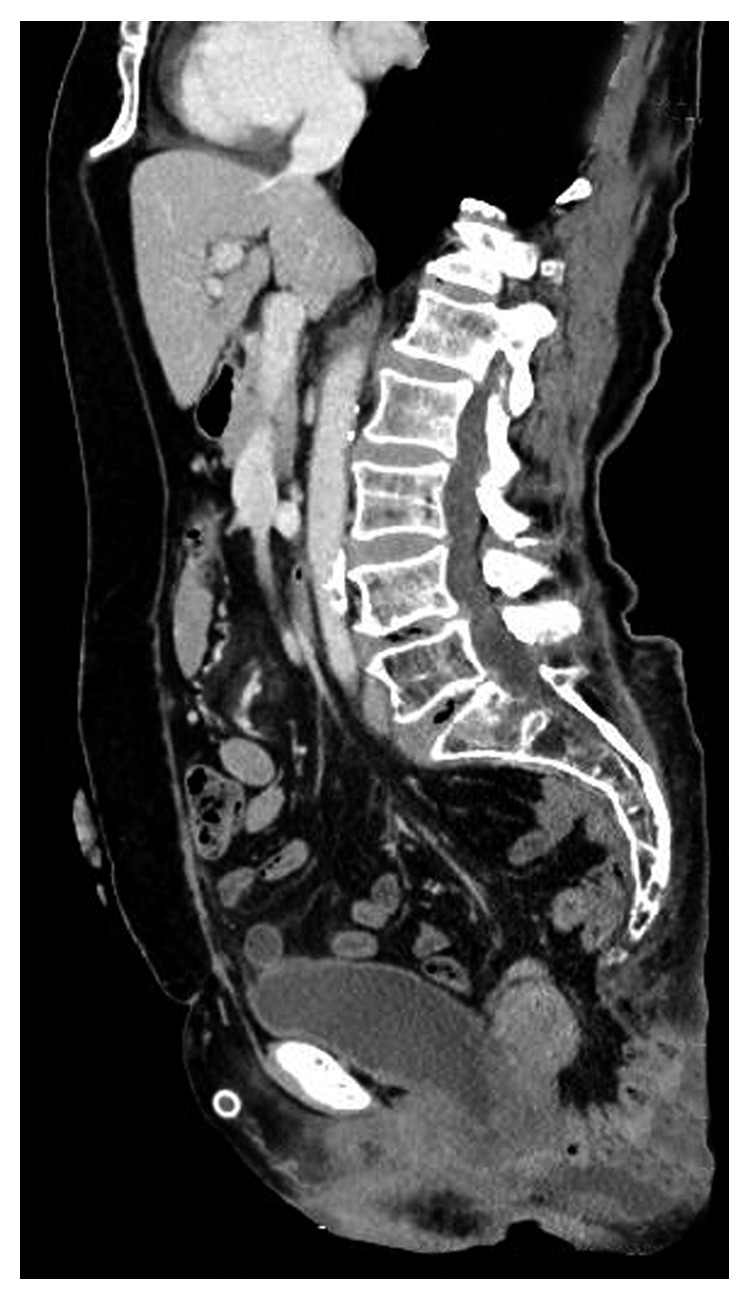
Herniated small bowel segments through the pelvis in CT scan.

A 67-year-old female patient with a locally advanced distal rectal adenocarcinoma (cT3N+) underwent laparoscopic abdominoperineal resection after short-term radiotherapy. A vaginal resection or repair was not required for the patient as she did not have any intraoperative problems. The patient with the pathology test result of pT2N0 received adjuvant chemotherapy. No complication was observed in the third and sixth month controls; however, the perineal hernia was detected at the ninth month. The abdominal computed tomography (CT) imaging taken without valsalva maneuver revealed no pathological findings except perineal hernia (Figure 1). The patient was, then, scheduled for follow-up, since she had no complaints. However, surgery was decided for the perineal hernia in the first postoperative year upon the growth of hernia and the restriction of daily activities due to the hernia.

The patient was placed on a Jackknife position. The perineal defect was revealed by dissecting the hernia vesicle through posterior approach. After the hernia content was reduced into the abdomen, the double-sided synthetic patch was placed over the defect and fixed to the levator ani muscle laterally, the vagina anteriorly, and the coccyx posteriorly with the 2/0 polyprolene sutures. The patient was discharged without any complication.

**Figure 2:**
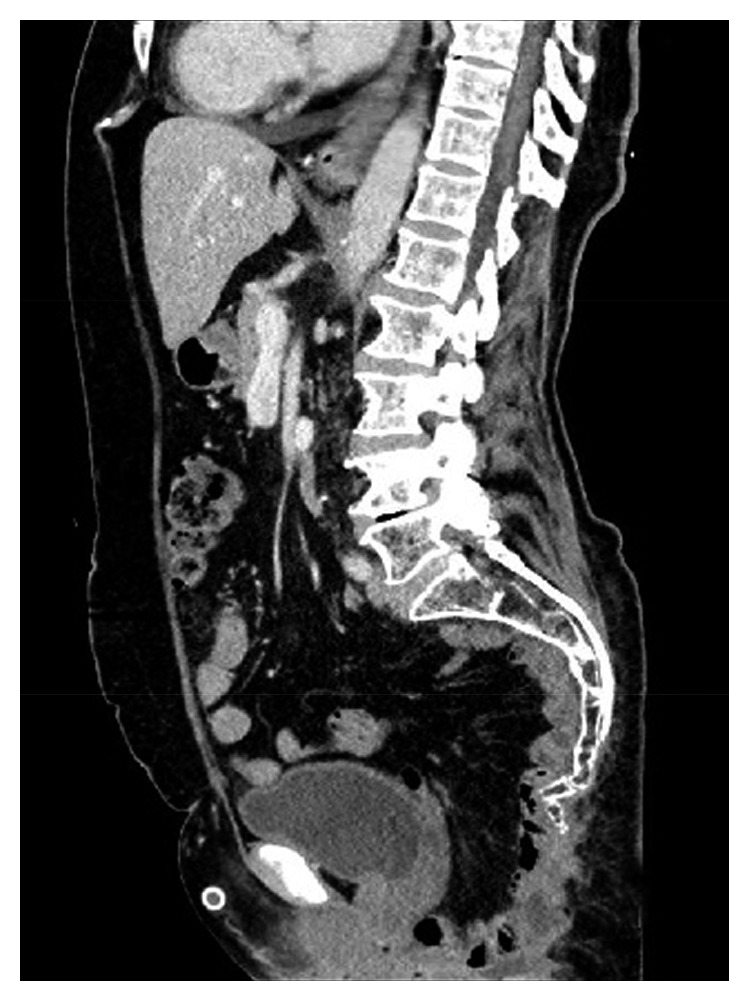
The sagittal image of the pelvis after perineal hernia repair.

**Figure 3:**
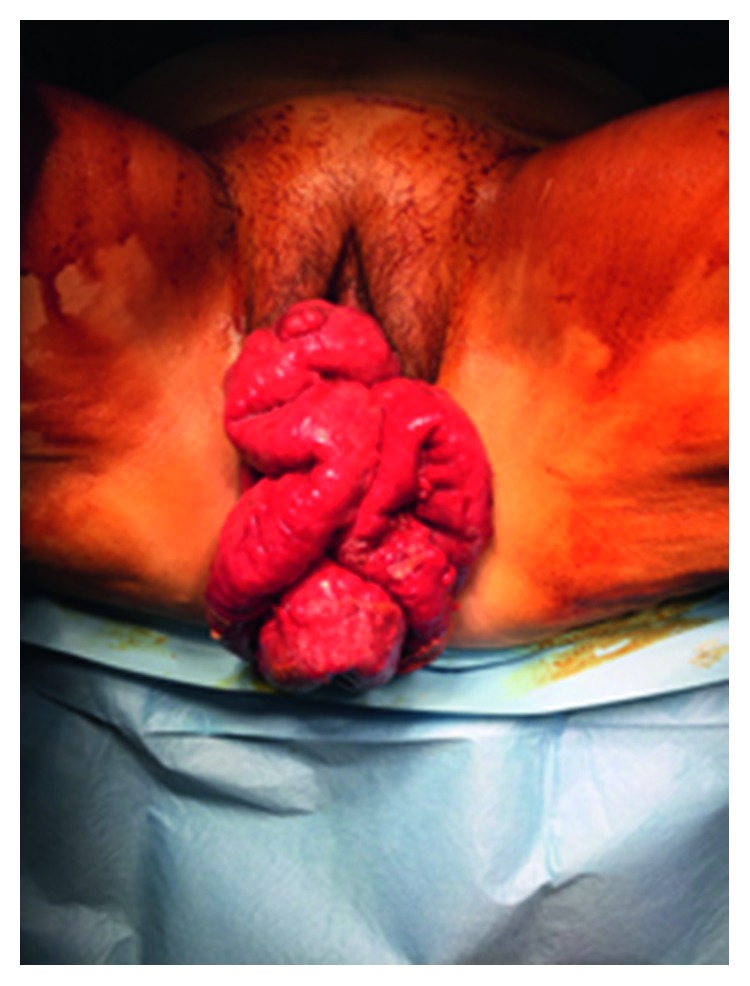
Vaginal evisceration of the small bowels.

**Figure 4:**
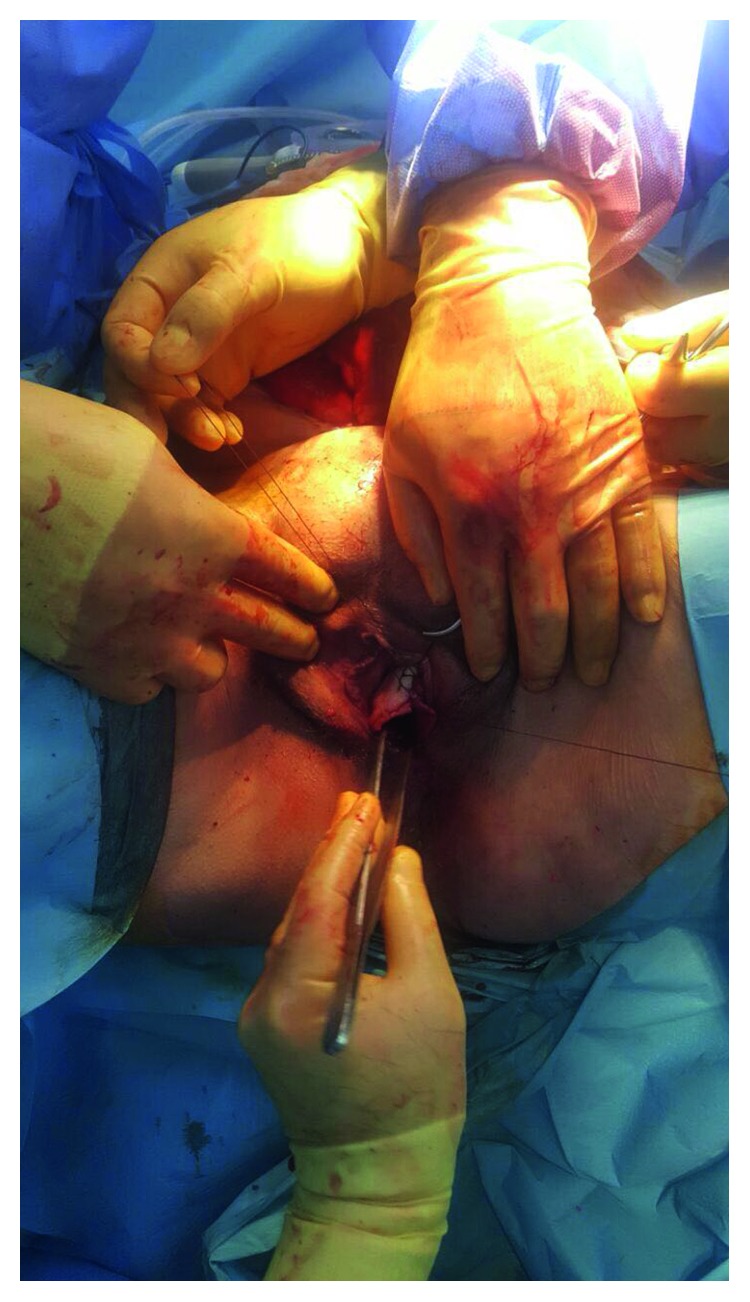
Repair of the vaginal defect with primary sutures.

The patient had no complaints related to the perineal hernia during the follow-up using CT imaging (Figure 2). The patient who was followed in the oncology clinic without problem was admitted to the emergency department with the complaint of prolapsed intestines from the vagina 12 months after the repair of the perineal hernia. During the examination, the small intestines were observed to be freely prolapsed from the vagina and several local ischemic changes were detected (Figure 3). In the emergency laparotomy, the terminal ileum revealed a herniation out of the posterior vaginal wall of the 110 cm ileum segment from the 10th cm of caecum. The ischemic areas were observed to be developed locally in the ileum. Following the withdrawal of the small intestine into the abdomen, a functional side-by-side ileocolic anastomosis was performed by resecting the ischemic segment with the caecum. The formerly placed mesh material was explored and seen as intact (no folding or detachment) and well placed below the levator ani muscle. As the formerly placed mesh material was located below levator ani muscle level, it was not covering the posterior vaginal wall. Instead, the previous mesh was only anchored to vagina anteriorly below the levator level. The vaginal defect was reconstructed with interrupted sutures by using the 3/0 polydioxanone (Figure 4). A new double-sided mesh material with antiadhesion barrier site facing the viscera was placed over the pelvic brim and fixed to the pubic bone anteriorly, the sacral promontory posteriorly, and the pelvic wall laterally by using the 2/0 polypropylene sutures. The patient was uneventfully discharged on the postoperative day 4; the wound sites were observed to be healed at three months, and no herniation finding was observed.

## 3. Discussion

The perineal herniation has been reported as 1–13% after the classical APR surgery [7]. The large pelvis, the previous hysterectomy, the radiation treatment, the excessive length of the small bowel mesentery, and the perineal infection are reported as the risk factors [8]. In addition, the dissection of Denonvilliers' fascia in men and the rectovaginal septum in women is thought to cause herniation [9]. Akatsu at al. speculated that the laparoscopic surgery may facilitate the small bowels sliding down to the pelvis due to the fewer intra-abdominal adhesions and may result in herniation [10].

The perineal herniation may be reconstructed through transabdominal or transperineal approach. Although the transperineal approach is a less invasive procedure, there are some disadvantages such as the poor exposure, the inadequate mobilization of the muscles, and the difficulty in competing with the adhesions compared with the transabdominal approach [11]. In addition, local, regional, or distal pedunculated muscle flaps may be used in reconstruction of the perineal defects. The vertical rectus abdominis myocutaneous flap (VRAM) is a common technique used in reconstruction of the perineal defects. VRAM flap has advantages such as long pedicle, large volume and surface area, and low incidence of necrosis [12].

In this case, a recurrent perineal herniation through vagina has occurred, and a portion of the small intestine eviscerated and strangulated after one year of the transperineal hernia repair following the abdominoperineal resection. It may be claimed that the transvaginal herniation occurred due to the chronic protrusion of the abdominal viscera to the weakened vaginal wall due to the loosening of the pelvic structures, the advanced age, and the previous surgeries. The intraoperative findings such as well-placed previous mesh material far from current vaginal defect and no relevant intra-abdominal adhesions may support the reason mentioned above. However, the anchoring suture placed in the vagina in the former perineal repair may be another reason for transvaginal herniation.
